# Non-Starch Polysaccharides in Wheat Beers and Barley Malt beers: A Comparative Study

**DOI:** 10.3390/foods9020131

**Published:** 2020-01-27

**Authors:** Miaomiao Li, Jinhua Du, Yaxin Zheng

**Affiliations:** College of Food Science and Engineering, Shandong Agricultural University, Tai’an, Shandong 271018, China; Limiaomiao0538@163.com (M.L.); saberzheng@hotmail.com (Y.Z.)

**Keywords:** non-starch polysaccharides, arabinoxylan, arabinogalactan, mannose polymers, β-glucan, wheat beers, barley malt beers, viscosity

## Abstract

Non-starch polysaccharides (NSPs) in beers attract extensive attention due to their health benefits. The aim of this work was to investigate and compare NSPs including arabinoxylan, arabinogalactan, β–glucans, and mannose polymers in wheat and barley malt beers as well as the influence on its quality. NSPs in wheat beers (1953–2923 mg/L) were higher than that in barley malt beers (1442–1756 mg/L). Arabinoxylan was the most abundant followed by arabinogalactan. In contrast to barley malt beers, wheat beers contained more mannose polymers (130–182 mg/L) than β-glucan (26–99 mg/L), indicating that more arabinoxylan, arabinogalactan, and mannose polymers came from wheat malt. The substitution degree of arabinoxylan in wheat beers (0.57–0.66) was lower than that in barley malt beers (0.68–0.72), while the degree of polymerization (38–83) was higher (*p* < 0.05) than that in barley malt beers (38–48), indicating different structures of arabinoxylan derived from barley malt and wheat malt. NSPs, especially arabinoxylan content, positively correlated (*p* < 0.01) with real extract and viscosity of beers. Furthermore, wheat and barley malt beers were well separated in groups by principal component analysis.

## 1. Introduction

As major component of dietary fibers, non-starch polysaccharides (NSPs) are vital structural elements of the cell wall of cereal endosperm as well as the aleurone layer, bran, and husk [1]. Among all these NSPs, arabinoxylan (AX), arabinogalactan, β-glucan, and mannose polymers are of considerable importance for human health [2]. Their quantity and molecular structure depend on the grain species, varieties, anatomic locations, and environmental conditions [3]. It was reported that the cell wall of barley starchy endosperm contained 75% (1,3;1,4)–β−D–glucan, 20% AX, 2% cellulose, and 2% glucomannan while the cell wall of the aleurone consisted of 71% AX, 26% (1,3;1,4) –β−D–glucans, and 3% cellulose and mannose polymers [4]. In contrast, the cell wall of wheat endosperm is principally composed of 65% feruloylated AX, 25% (1,3;1,4)–β–D–glucan, 7% glucomannan, and 3% cellulose [5].

AX is composed of a chain of β–D–xylopyranosyl residues, connected by β–1,4–glycosidic linkages, being either non-substituted or substituted with α–L–arabinofuranose at the C3 or C2 positions or at both in varying patterns [6]. The (1,3;1,4)–β–glucans are unbranched chains of D–glucopyranose residues with β–(1,4) linkages and β–(1,3) linkages in ratios ranging from 3.2:1 to 6.6:1 [7]. The arabinogalactan-peptide of wheat consists of predominant polysaccharides (92%) and peptide materials, which is composed of 15–20 amino acids, and the carbohydrate moiety is covalently associated with peptide chain through the hydroxyproline residues [8]. Fincher et al. [9] found that the arabinogalactan was composed of a β–D–galactopyranosyl backbone substituted with α–L–arabinofuranosyl residues and with an arabinose/galactose ratio of 0.66–0.73.

NSPs from cereal sources have been reported to impart health benefits [10]. Extensive studies focused mainly on the structural characteristics and properties of AX [11], β–glucan [12], and arabinogalactan-peptide [13] from various cereals. As the most important NSPs, AX and β–glucan have attracted extensive attention due to their increasing functional properties, including immunomodulatory properties [14], glycemic-reducing effect [15], cholesterol-lowering effect [16], antioxidant activity [17], and prebiotic activity [18]. Mannose polymers have also been reported to exhibit physiological activities and functions [19].

In beer brewing, AX and β–glucan can provoke filtration problems such as low extract yield, high wort viscosity, decreased filtration rate, and haze formation during beer storage [20]. Previously, these filtration problems were mainly attributed to β–glucan from barley, which might increase the viscosity of beer by forming gels consisting primarily of β–glucan molecules with high molecular weights [21]. However, insufficient breakdown of AX might also cause filtration problems. Some AX were solubilized from the cell wall of barley but were not extensively degraded by endogenous enzymes during malting [22]. A high proportion of ferulic acid dimers detected in the water-extractable AX from malt suggested that AX might be partially cross-linked with the possibility to cause filtration problems [23]. These brewing problems would be more pronounced when wheat malt was used due to higher content and higher molecular weight of AX.

As far as we know, except for AX and β–glucan, little attention has been devoted to mannose polymers and arabinogalactan and to their influence on beer and beer brewing. Therefore, the aim of this work was to analyze and compare the differences of NSPs in wheat beers and barley malt beers, including AX, arabinogalactan, β–glucans, and mannose polymers, and to uncover their relationship with physicochemical indices of beer and their influence on beer quality.

## 2. Materials and Methods

### 2.1. Materials and Reagents

A total of twenty-one commercial beer samples were purchased from local RT-Mart in Tai’an, and Jingdong online mall, China, including fifteen wheat and six barley malt beers. Amyloglucosidase (Enzyme Commission (EC) number 3.2.1.3) and β-glucan assay kit (K-BGLU) were purchased from Megazyme International (Bray, Ireland). Monosaccharide standards including L–arabinose, D–xylose, D–galactose, D–mannose, and D–glucose were obtained from Sigma-Aldrich Co. (Saint Louis, Missouri, USA). All other chemicals such as phenol, sulfuric acid, ammonium hydroxide, sodium borohydride, trifluoroacetic acid, 1–methylimidazole, acetic anhydride, and dichloromethane were of at least analytical grade.

### 2.2. Determination of Main Physicochemical Indices, Total Carbohydrate, and β-Glucans

The original extract and real extract of beers were determined according to European Brewery Convention (EBC) Analytic Method 9.4. (Brussels, Belgium) Alcohol content and degree of fermentation were evaluated using EBC Analytic Method 9.2.6 and 9.5. Viscosityof beer was measured by a Haake Falling Ball viscometer (Thermo Scientific, Bremen, Germany) as stated by EBC Analytic Method 9.38 (Brussels, Belgium). Total carbohydrate content was estimated by spectrophotometry according to American Society of Brewing Chemists (ASBC) Analysis Method (7.41.1) (St. Paul, MN, USA). β-Glucan content was detected with a mixed-linkage β-glucan assay kit (K-BGLU) (Megazyme, Bray, Ireland) in light of the Association of Official Analytical Chemists (AOAC) Method 995.16 (Rockville, MD, USA).

### 2.3. Determination of Total NSPs, Arabinoxylan, Arabinogalactan, and Mannose Polymers

#### 2.3.1. Purification of NSPs in Beers

**Figure 1:**
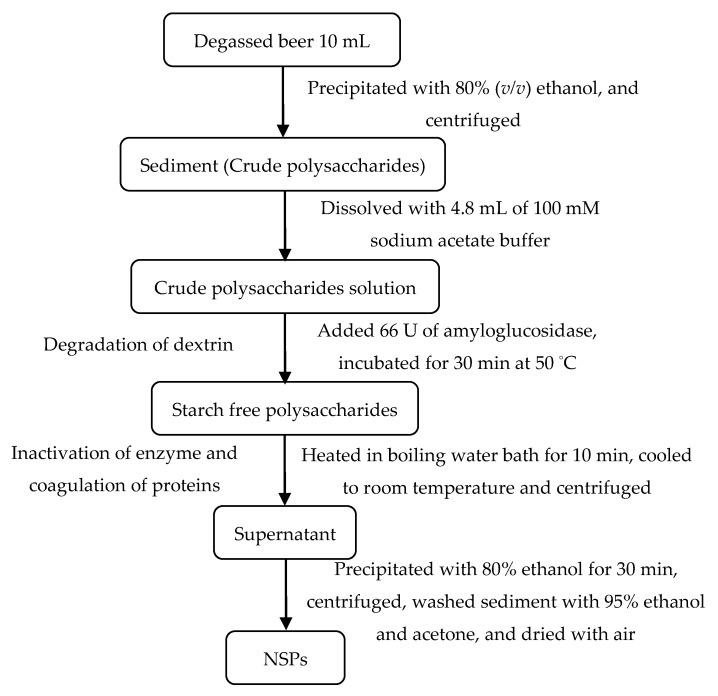
Purification procedure of non-starch polysaccharides (NSPs) in commercial beers.

The purification procedure of NSPs in beers is shown in Figure 1 and refers to our previous study [24]: 40 mL of ethanol was gradually added to 10 mL of degassed and centrifuged beer to mix thoroughly and stand overnight at 4 °C and then centrifuged at 5000 g for 10 min. After discarding the supernatant, the sediment was dissolved with 4.8 mL of sodium acetate buffer (100 mM, pH 5.0). Subsequently, 66U amyloglucosidase was mixed with the solution and incubated in 50 °C water bath for 30 min to degrade dextrins. It was then cooled to room temperature and centrifuged after heating in boiling water bath for 10 min to inactivate the enzyme and to coagulate the soluble proteins. The supernatant was precipitated with ethanol to a final concentration of 80% and centrifuged at 5000× *g* for 10 min after standing for 30 min. The sediment was washed with 95% ethanol and acetone once and then air dried.

#### 2.3.2. Monosaccharide Composition

Monosaccharide composition analysis was performed as reported by Li et al. [24]. Briefly, the purified NSPs from beers were dissolved and hydrolyzed to monosaccharides with 2.0 M trifluoroacetic acid. The alditol acetates of monosaccharides were prepared by reduction with sodium borohydride and derivatization with acetic anhydride. To analyze the reducing end of sugar content, the reduction was performed prior to hydrolysis and derivatization. Alditol acetates were extracted with dichloromethane and injected into a Shimadzu GC-2010 Plus gas chromatograph (Kyoto, Japan) and then separated on a column DM-2330 (L × I.D. 30 m × 0.32 mm, d_f_ 0.2 μm) (DIKMA, Beijing, China). The calibration curves of standard monosaccharides were prepared with concentration of corresponding alditol acetates and the peak area. The square of determination coefficient (*r*^2^) was at least 0.9992, as shown in Table 1.

#### 2.3.3. Calculation

Total NSP content was calculated using alditol acetate contents of monosaccharides including L-arabinose (Ara), D-xylose (Xly), D-galactose (Gal), D-mannose (Man), and D-glucose (Glc) determined by gas chromatography (GC) method according to Equation (1). Factors 0.88 and 0.90 were used to standardize incorporation of water during hydrolysis of pentose and hexose. AX content was estimated by Equation (2) and corrected for arabinose bound in arabinogalactan-peptide (0.7 × Gal). The degree of substitution of AX was expressed as the ratio of arabinose to xylose (A/X), which was estimated by Equation (3). The average degree of polymerization (avDP) of AX was estimated by Equation (4). Reduced xyl is alditol acetate content of xylose determined by GC after reduction followed by hydrolysis and derivatization, as stated in 2.3.2. The polysaccharide component of arabinogalactan-peptide was given as arabinogalactan [9] by Equation (5), which was calculated as a ratio of 0.70:1 for arabinose to galactose [8]. Mannose polymers content was calculated as mannan according to Equation (6).

NSPs (mg/L) = 0.9 × (Glc + Man + Gal) + 0.88 × (Xyl + Ara)(1)

AX (mg/L) = 0.88 × (Ara − 0.7 × Gal + Xyl)(2)

A/X = (Ara − 0.7 × Gal)/Xyl(3)

avDP (AX) = (Ara − 0.7 × Gal + Xyl)/Reduced Xyl(4)

Arabinogalactan (mg/L) = 0.9 × Gal + 0.88 × 0.7 × Gal(5)

Mannan (mg/L) = 0.9 × Man(6)

### 2.4. Statistical Analysis

All data were the mean value of at least three measurements. Data were processed using SPSS Statistics 22 (SPSS Inc., Chicago, IL, USA). Statistical analyses of the differences between the parameters of the two types of beers were evaluated by analysis of variance (ANOVA) and Tukey’s test (*p* < 0.05). Factor analysis was used to perform principal component analysis (PCA), and data were analyzed by Pearson correlation (double-tailed test); correlation under *p* < 0.05 was considered to be significant.

## 3. Results and Discussion

### 3.1. Total Carbohydrate and Non-Starch Polysaccharides

Table 2 shows the main physicochemical indices of beers. In wheat beers, total carbohydrate ranged from 27.1 to 39.1 g/L and real extract was in the range 3.64–4.36% (*w/w*), while total carbohydrate ranged from 30.6 to 33.9 g/L and real extract was in the range 3.46–4.16% (*w/w*) in barley malt beers. There were no significant differences (*p* > 0.05) in physicochemical indices of original extract, real extract, total carbohydrate, alcohol by volume, and real degree of fermentation between wheat and barley malt beers. However, the viscosity showed a significant difference among them; it was much lower for barley malt beers than wheat beers, which was consistent with the level of NSPs in them.

As exhibited in Table 2, the total NSP content was in the range of 1953–2923 mg/L in wheat beers and of 1442–1756 mg/L in barley malt beers. The average content was dramatically higher (*p* < 0.05) in wheat beers (2355 ± 235 mg/L) than in barley malt beers (1565 ± 105 mg/L). The average proportion of NSPs in total carbohydrate was 6.8 ± 0.5% (*w/w*) in wheat beers, also significantly higher (*p* < 0.05) compared to 4.8 ± 0.3% (*w/w*) in barley malt beers. The higher presence of soluble NSPs in wheat beers was attributed to the raw material of wheat malt, since barley malt beers were made from pure barley malt while wheat beers were made from barley malt and wheat malt. As reported by Suhasini et al. [25], water soluble NSP content of wheat malt was 5.43%, higher than in wheat (4.93%). In contrast, Cyran et al. [20] reported that water-extractable NSPs constituted 1.39% of barley malt grist.

### 3.2. Individual Non-Starch Polysaccharide

#### 3.2.1. Arabinoxylan (AX)

##### AX Content

AX content was in the range of 1271–1951 mg/L in wheat beers and of 790–1000 mg/L in barley malt beers. The average level of AX in wheat beers was 1491 ± 184 mg/L, almost twice as much as that in barley malt beers (865 ± 78 mg/L) (Table 3). Similar to the study of Courtin et al. [26], AX content was in the range of 1.64–1.78 g/L in German wheat beers and of 0.63–1.04 g/L in lager beers. In the analysis of forty German wheat beer samples, AX content was between 0.87 g/L and 2.88 g/L [27]. However, Schwarz and Han [28] found that the AX level in three German wheat beers was in the range of 3.10–4.21 g/L and much higher than that in our analysis, which might be attributed to interference of unpurified samples and unincorporated arabinose bound to arabinogalactan-peptide.

The much higher level of AX in wheat beers was due to a higher level of soluble AX in wheat malt than that in barley malt. The particularly high AX level in WB15 was probably due to the high proportion of wheat malt utilized. As determined by Fincher and Stone [4], AX content of wheat malt and dehusked barley malt were 12.6% and 3.1–4.0%, respectively. However, Li et al. [29] reported that total AX was 6.90% in wheat malt and in the range of 3.64–6.40% in six barley malts and that water extractable AX were 0.98% and 0.42–0.70%, respectively. During the malting process, water-extractable AX content in wheat was increased by 97% [27].

##### A/X and avDP of AX

The A/X ratio is a direct measure for the degree of substitution and an important indicator for structural feature of AX molecules [30]. As indicated in Table 4, A/X ranged from 0.57 to 0.66 in wheat beers and from 0.68 to 0.72 in barley malt beers; the average value in wheat beers (0.59 ± 0.02) was significantly lower (*p* < 0.05) than that of barley malt beers (0.70 ± 0.02), suggesting different structural features of AX from wheat malt and barley malt. Courtin et al. [26] found that A/X fell within a range of 0.49–0.57 in wheat beers and of 0.54–0.66 in lager beers. However, A/X of German wheat beers determined by Krahl et al. [27] was between 0.69 and 0.80, which was higher than that of wheat beers in our analysis. The different molecular structure depends on the grain species, varieties, and anatomic location and is affected by environmental conditions [3]. As previously reported, for water-extractable AX from wheat, A/X value was typically from 0.50 to 0.60 [30], and a similar value of 0.61 ± 0.02 was also obtained by Buksa et al. [31]. According to the work of Comino et al. [32], A/X of water-extractable AX from the endosperm flour of wheat and hull-less barley were 0.58 and 0.64, respectively.

As indicated in Table 4, avDP of AX was in the range of 38–83 in wheat beers, including ten samples ranging from 50 to 64, four ranging from 38 to 44 and one sample reaching the maximum of 83, whereas it was only in the range 38–48 in barley malt beers. Their mean values were 54 ± 12 and 43 ± 4, respectively; a significant difference (*p* < 0.05) was found among them. The molecular mass calculated by avDP was in the range of 4770–10,974 Da in wheat beers and of 5034–6354 Da in barley malt beers. Higher avDP in wheat beers appeared to be attributed to the use of wheat malt [33]. AX in wheat beers with lower A/X and higher avDP might show different physical properties from that in barley malt beers, thus potentially affecting the viscosity, turbidity, and even mouthfeel of beers.

#### 3.2.2. Arabinogalactan

Arabinogalactan content was in the range of 273–393 mg/L in wheat beers and significantly higher (*p* < 0.05) than that in barley malt beers (189–211 mg/L) (Table 3). On the other side, the arabinogalactan peptide content was in the range of 0.09–0.18 g/L in larger beers and of 0.31–0.37 g/L in wheat beers, as reported by Courtin et al. [26]. Furthermore, arabinogalactan level was typically a fifth to a third of the AX level. Higher arabinogalactan level in wheat beers indicated higher soluble fractions in wheat malt. AX and highly branched arabinogalactan were typically co-extracted with water in wheat [34]. As previously reported, the arabinogalactan content was in the range of 0.24%–0.33% in wheat endosperm flour [35] and of 0.3–0.4% in wheat flour [36]. However, it ranged from 0.47% to 0.93% in eight spring whole wheat flour [13].

#### 3.2.3. β-Glucan

As demonstrated in Table 3, β-glucan content was in the range 26–99 mg/L in wheat beers, which was much lower (*p* < 0.05) than that in barley malt beers (106–190 mg/L). Similar content obtained by Schwarz and Han [28] was in a range of 21.4–57.2 mg/L in three wheat beers. Higher level in barley malt beers was attributed to β-glucan, which was abundant in barley malt. Barley contained the highest level of β-glucan (3–20%) in cereals [37]. Henry [38] reported that β-glucan contents in wendosperm of wheat and barley were 0.3% and 4.1%, respectively, While β-glucan accounted for 0.50% of barley malt [20] and 0.27% of wheat malt [28].

#### 3.2.4. Mannose Polymers

Mannose was observed in monosaccharides composition of NSPs; therefore, it existed as polymers. The mannose polymers content expressed as mannan was in the range of 130–182 mg/L in wheat beers and of 119–145 mg/L in barley malt beers. The average content of mannan in wheat beers (157 ± 15 mg/L) was slightly higher (*p* < 0.05) than in barley malt beers (130 ± 10 mg/L) (Table 3). Thus, it was speculated that the mannose polymers in beers were derived from the brewing grains such as barley and wheat. Voragen et al. [22] reported that mannose accounted for 9% and 4% of sugar composition of total NSP in dehusked barley and malt, respectively, and that mannose increased from 3% to 25% in water soluble fraction during malting. In previous study, mannose constituted 2–3% of water-extract fraction from barley endosperm flours [39] and 2.7% and 3.6% monosaccharide composition of the insoluble NSPs from barley hull-less endosperm flour and wheat endosperm flour, respectively [32]. All these documents explicitly reflected the presence of mannose polymers, which were speculated originating from cell wall components.

### 3.3. Percentage of Arabinoxylan, Arabinogalactan, β-Glucan, and Mannose Polymers in NSPs

As shown in Table 3, AX was the most abundant NSP in beers, accounting for 58.8–71.0% in wheat beers and 51.9–57.8% in barley malt beers. The following was arabinogalactan, accounting for 13.2–15.5% of NSPs in wheat beers and 11.4–13.6% in barley malt beers. They were both significantly higher (*p* < 0.05) in wheat beers than in barley malt beers. For wheat beers, the percentage of mannan in NSPs was 5.4–8.3% higher than that of β-glucan (1.2–4.5%). However, β-glucan accounted for 6.0–12.5% of NSPs in barley malt beers, which was higher than that of mannan (7.5–9.4%). The sum of AX, arabinogalactan, β-glucan, and mannan accounted for 80.6–93.5% of total NSP in wheat beers while 82.2–87.5% were in barley malt beers, less than 100% of the total NSP. The reason was that there was still a portion of glucose in monosaccharide composition of NSPs besides β-glucan, which was supposed to be from glucomannan—another possible mannose polymer in beers except for mannan. The details have been discussed in our recent report [24].

AX was the major component of NSPs in both wheat beers and barley malt beers. Arabinose and xylose accounted for 70% sugar composition of total NSP in pilot-brewed barley malt beers [40] and 65% monosaccharide composition of the water-extractable NSPs in malting barley [20]. By contrast, as the main constituent of wheat grain cell walls, AX also made up approximately 64% of NSPs in wheat [41]. In summary, AX was the main dietary fibers in beers and plays an important role in beer and beer brewing.

### 3.4. Principal Component Analysis of NSP Contents and Physicochemical Indices

PCA was applied to the eleven original variables of all beer samples; these variables included NSP indicators and physicochemical indices. NSP indicators included contents of AX, arabinogalactan, β-glucans, mannose polymers, and total NSP, while physicochemical indices included the original extract, real extract, total carbohydrates, alcohol, real degree of fermentation, and viscosity.

The correlation coefficients of these original variables are presented in Table 5. Each NSP, total NSP, and total carbohydrate showed no significant correlation (*p* > 0.05) with the original extract and alcohol. AX, arabinogalactan, and total NSP contents were positively correlated (*p* < 0.01) to the real extract, while β-glucan and mannose polymers contents had no significant correlation. The highly positive correlation coefficient (*p* < 0.01) between total carbohydrates and real extract suggested that carbohydrates were the main component of real extract in beers. NSP contents correlated positively (*p* < 0.01) with beer viscosity except for β-glucan content, and the correlation coefficients of each NSP and total NSP with beer viscosity were higher than that of total carbohydrate (*p* < 0.05). It indicated that, although NSPs accounted for only a small proportion of total carbohydrate, they played an important role in the viscosity of beer, especially for AX. Total carbohydrate in beer negatively correlated (*p* < 0.01) with the real degree of fermentation; it was not difficult to understand that higher fermentation degree meant lower residual carbohydrates. However, no significant correlation (*p* > 0.05) was found between NSP content and the fermentation degree because of the fact that NSPs could not be utilized during beer fermentation. Contents of AX, arabinogalactan, β-glucan, and mannose polymers were positively correlated (*p* < 0.01) with each other except for β-glucan, which was negatively correlated with other NSPs and total NSP content. This was consistent with the results that there was lower amount of β-glucan but higher amount of other NSPs and total NSP in wheat beers, which was opposite to that in barley malt beers.

NSPs, especially AX content, were positively correlated with viscosity of beers. Water soluble NSPs can increase the viscosity; can improve the foam stability; and can increase the taste, flavor, and mellowness of beers, which are beneficial for improving beer quality. High viscosity caused by high molecular weight AX and other NSPs may also reduce filtration performance in the beer brewing process [27]. Therefore, water-soluble NSPs play an important role in beer quality and beer brewing.

Table 6 shows the rotated component loadings of the original variables and evaluating indices. It was noted that the first three principal components (PCs) accounted for 88.1% of the total variation. The first principal component PC1 accounted for 36.5% of the total variation. It was loaded heavily on the contents of total NSP, AX, arabinogalactan, mannose polymers, and β-glucan (negative loading) as well as on little contributions from other variables, indicating the importance of NSPs in beers. The second principal component PC2, accounting for 27.4% of the total variation, was loaded heavily on the indices of real extract and total carbohydrates. The indices of original extract, alcohol, and viscosity of beers were observed to have high loadings for the third principal component PC3, which explained 24.2% of the variation.

**Figure 2:**
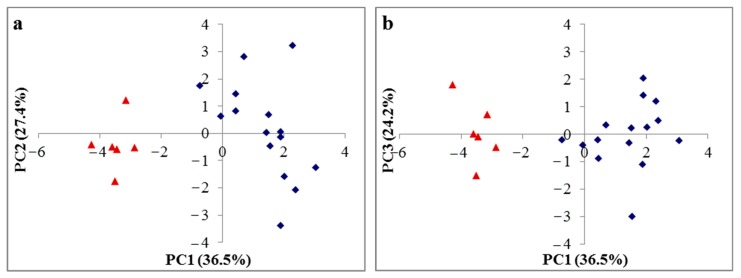
Principal component scores scatter plot for 21 beer samples: (**a**) Score scatter plots of PC1 vs. PC2 and (**b**) score scatter plots of PC1 vs. PC3. For illustration purposes, blue rhomb represents wheat beers and red triangle represents barley malt beers.

Figure 2 shows the component scores plot for 21 beer samples on the first two component axes (Figure 2a) and on the first and third component axes (Figure 2b). All beer samples were clustered into two clusters in the score scatter plots; one cluster was wheat beers, and another was barley malt beers. Most wheat beers had a positive score on the first principal component, while barley malt beers had a negative score. Generally, good classification of beers was obtained using the first principal component.

## 4. Conclusions

Total NSP content and its percentage in total carbohydrate were higher in wheat beers than in barley malt beers. Mannose polymers were found in beers and expressed as mannan. The level of AX, arabinogalactan, and mannose polymers were significant higher in wheat beers, while β-glucan content was significant higher in barley malt beers, indicating higher potential soluble AX, arabinogalactan, and mannose polymers in wheat malt than in barley malt. In wheat beers, AX was the most abundant NSP, followed by arabinogalactan, mannose polymers, and β-glucan, indicating the importance of AX in wheat beer and beer brewing. A/X ratio was lower while avDP was higher in wheat beers than in barley malt beers, indicating different structural features of AX from wheat and barley malt. NSPs, especially AX content, positively correlated with the real extract and viscosity of beers. Furthermore, wheat beers and barley malt beers were well separated by PCA analysis. Further studies should be performed to elucidate the molecular structures and functional properties of NSPs in beers. In particular, the effects of NSPs on the filtration and brewing processing and on nutritional and nutraceutical properties beneficial to human health.

## Figures and Tables

**Table 1:** Calibration curves of standard monosaccharides.

Monosaccharides	Retention Time (min)	Calibration Curves	Determination Coefficient (*r*^2^)	Linearity Range (mg/L)	LOD (mg/L)	LOQ (mg/L)
L-arabinose	6.969	Y = 2158.161x + 171.123	0.9997	0-100	0.54	1.79
D-xylose	8.339	Y = 1873.081x + 80.836	0.9992	0-200	0.73	2.43
D-mannose	11.453	Y = 1586.438x − 639.508	0.9994	0-50	1.33	4.43
D-galactose	12.577	Y = 1762.792x − 585.619	0.9994	0-50	1.29	4.29
D-glucose	13.671	Y = 1352.029x − 883.032	0.9994	0-100	1.84	6.14

Note: ***r^2^***, square of the determination coefficient; LOD, limit of detection; LOQ, limit of quantitation.

**Table 2:** Overview of beer main physicochemical indices, total carbohydrate, and total non-starch polysaccharides.

	OE	RE	ABV	Viscosity	RDF	TC	NSPs	NSPs/TC
BB1	10.4 ± 0.0	3.46 ± 0.00	4.50 ± 0.01	1.33 ± 0.01	67.9 ± 0.0	30.6 ± 0.6	1520 ± 3	5.0 ± 0.0
BB2	11.3 ± 0.0	3.81 ± 0.00	4.90 ± 0.00	1.39 ± 0.00	67.6 ± 0.1	32.2 ± 0.5	1548 ± 5	4.8 ± 0.0
BB3	11.4 ± 0.0	3.74 ± 0.00	5.01 ± 0.00	1.39 ± 0.00	68.5 ± 0.0	32.6 ± 1.6	1588 ± 2	4.9 ± 0.0
BB4	11.5 ± 0.0	3.78 ± 0.00	5.05 ± 0.00	1.37 ± 0.00	68.4 ± 0.0	33.2 ± 0.4	1442 ± 14	4.3 ± 0.0
BB5	12.2 ± 0.1	4.16 ± 0.01	5.27 ± 0.01	1.46 ± 0.00	67.2 ± 0.1	33.9 ± 0.8	1756 ± 8	5.2 ± 0.0
BB6	12.3 ± 0.0	3.84 ± 0.00	5.58 ± 0.01	1.39 ± 0.01	70.2 ± 0.2	32.1 ± 1.1	1536 ± 5	4.8 ± 0.0
Mean (*n* = 6)	11.5 ± 0.7 ^a^	3.80 ± 0.22 ^a^	5.05 ± 0.36 ^a^	1.39 ± 0.04 ^b^	68.3 ± 1.1 ^a^	32.4 ± 1.1 ^a^	1565 ± 105 ^b^	4.8 ± 0.3 ^b^
WB1	10.3 ± 0.0	3.87 ± 0.00	4.19 ± 0.00	1.42 ± 0.00	63.7 ± 0.0	32.7 ± 1.5	2145 ± 17	6.5 ± 0.1
WB2	11.2 ± 0.0	3.91 ± 0.01	4.76 ± 0.00	1.46 ± 0.00	66.4 ± 0.0	35.6 ± 0.6	2324 ± 5	6.5 ± 0.0
WB3	11.4 ± 0.0	3.99 ± 0.00	4.84 ± 0.00	1.47 ± 0.00	66.3 ± 0.0	35.3 ± 0.3	2407 ± 8	6.8 ± 0.0
WB4	11.5 ± 0.0	3.93 ± 0.01	4.93 ± 0.01	1.49 ± 0.00	67.0 ± 0.1	36.3 ± 0.5	2194 ± 111	6.0 ± 0.0
WB5	11.6 ± 0.0	3.96 ± 0.00	5.00 ± 0.00	1.48 ± 0.00	67.2 ± 0.1	35.4 ± 1.1	2289 ± 8	6.5 ± 0.0
WB6	11.6 ± 0.0	3.85 ± 0.00	5.09 ± 0.00	1.42 ± 0.00	68.2 ± 0.0	34.1 ± 0.3	2382 ± 47	7.0 ± 0.1
WB7	11.6 ± 0.0	3.77 ± 0.00	5.16 ± 0.01	1.49 ± 0.00	68.9 ± 0.0	31.3 ± 0.3	2299 ± 1	7.3 ± 0.0
WB8	11.8 ± 0.0	4.14 ± 0.01	5.02 ± 0.00	1.51 ± 0.00	66.2 ± 0.2	36.3 ± 0.4	2179 ± 22	6.0 ± 0.1
WB9	11.8 ± 0.0	4.15 ± 0.00	5.02 ± 0.00	1.55 ± 0.00	66.2 ± 0.0	34.9 ± 0.8	2536 ± 9	7.3 ± 0.0
WB10	11.9 ± 0.1	3.99 ± 0.00	5.17 ± 0.00	1.49 ± 0.00	67.7 ± 0.0	37.9 ± 0.2	2417 ± 7	6.4 ± 0.0
WB11	12.0 ± 0.0	3.93 ± 0.01	5.31 ± 0.01	1.40 ± 0.00	68.7 ± 0.1	29.9 ± 0.6	2141 ± 18	7.2 ± 0.1
WB12	12.3 ± 0.0	3.64 ± 0.00	5.68 ± 0.00	1.56 ± 0.00	71.7 ± 0.2	27.1 ± 1.3	1953 ± 117	7.2 ± 0.1
WB13	12.3 ± 0.0	4.40 ± 0.00	5.24 ± 0.00	1.54 ± 0.00	65.8 ± 0.1	37.9 ± 1.4	2615 ± 11	6.9 ± 0.0
WB14	12.5 ± 0.0	4.00 ± 0.00	5.61 ± 0.01	1.52 ± 0.00	69.5 ± 0.0	34.9 ± 0.6	2514 ± 7	7.2 ± 0.0
WB15	12.5 ± 0.0	4.36 ± 0.00	5.39 ± 0.00	1.79 ± 0.00	66.7 ± 0.0	39.1 ± 0.3	2923 ± 12	7.5 ± 0.0
Mean (*n* = 15)	11.7 ± 0.6 ^a^	3.99 ± 0.20 ^a^	5.09 ± 0.36 ^a^	1.51 ± 0.09 ^a^	67.3 ± 1.9 ^a^	34.6 ± 3.2 ^a^	2355 ± 235 ^a^	6.8 ± 0.5 ^a^

OE, original extract (^o^P); RE, real extract (% *w/w*); ABV, alcohol by volume (%, *v/v*); Viscosity (mPa﹒s) RDF, real degree of fermentation (%); TC, total carbohydrate (g/L); NSPs, non-starch polysaccharides (mg/L); NSPs/TC, proportion of total NSP in total carbohydrate (%, *w/w*); BB, barley malt beers; WB, wheat beers. Values are mean ± standard deviation of three replications. Means represent average value ± standard deviation of all beer samples in the same category; different letters indicate significant difference among the average values of barley malt beers and wheat beers under the *p* < 0.05 level.

**Table 3:** Content and percentage of arabinoxylan, arabinogalactan, β-glucan, and mannose polymers in total non-starch polysaccharides.

Beer *No.*	Individual NSP Content (mg/L)				Percentage of Individual NSP in Total NSP (%, *w/w*)			
	AX	AG	BG	MP	AX	AG	BG	MP
BB1	790 ± 5	197 ± 4	190 ± 1	125 ± 1	51.9 ± 0.3	12.9 ± 0.3	12.5 ± 0.1	8.2 ± 0.1
BB2	895 ± 6	211 ± 5	113 ± 0	125 ± 7	57.8 ± 0.4	13.6 ± 0.3	7.3 ± 0.0	8.0 ± 0.4
BB3	881 ± 11	193 ± 4	128 ± 0	119 ± 2	55.4 ± 0.1	12.1 ± 0.3	8.0 ± 0.0	7.5 ± 0.1
BB4	814 ± 7	189 ± 3	125 ± 2	127 ± 4	56.4 ± 0.5	13.1 ± 0.2	8.7 ± 0.1	8.8 ± 0.2
BB5	1000 ± 3	200 ± 5	106 ± 2	138 ± 3	56.9 ± 0.2	11.4 ± 0.3	6.0 ± 0.1	7.8 ± 0.2
BB6	813 ± 4	200 ± 4	187 ± 0	145 ± 1	52.9 ± 0.3	13.0 ± 0.2	12.2 ± 0.0	9.4 ± 0.1
Mean (*n* = 6)	865 ± 78 ^b^	198 ± 8 ^b^	141 ± 37 ^a^	130 ± 10 ^b^	55.2 ± 2.3 ^b^	12.7 ± 0.8 ^b^	9.1 ± 2.6 ^a^	8.3 ± 0.7 ^a^
WB1	1369 ± 6	284 ± 6	47 ± 1	148 ± 5	63.8 ± 0.3	13.2 ± 0.3	2.2 ± 0.1	6.9 ± 0.2
WB2	1375 ± 8	342 ± 7	66 ± 4	168 ± 4	59.2 ± 0.3	14.7 ± 0.3	2.8 ± 0.2	7.2 ± 0.2
WB3	1538 ± 8	340 ± 5	72 ± 1	130 ± 2	63.9 ± 0.3	14.1 ± 0.2	3.0 ± 0.0	5.4 ± 0.1
WB4	1309 ± 8	306 ± 3	99 ± 2	156 ± 1	59.7 ± 0.4	13.9 ± 0.1	4.5 ± 0.1	7.1 ± 0.1
WB5	1379 ± 12	337 ± 7	66 ± 1	164 ± 7	60.2 ± 0.5	14.7 ± 0.3	2.9 ± 0.0	7.2 ± 0.3
WB6	1529 ± 33	370 ± 7	39 ± 0	166 ± 4	64.2 ± 1.4	15.5 ± 0.3	1.6 ± 0.0	7.0 ± 0.0
WB7	1458 ± 3	347 ± 3	92 ± 1	163 ± 1	63.4 ± 0.1	15.1 ± 0.1	4.0 ± 0.1	7.1 ± 0.0
WB8	1400 ± 17	330 ± 3	88 ± 1	137 ± 3	64.3 ± 0.8	15.1 ± 0.1	4.0 ± 0.0	6.3 ± 0.1
WB9	1490 ± 7	337 ± 3	76 ± 2	140 ± 4	58.8 ± 0.3	13.3 ± 0.1	3.0 ± 0.1	5.5 ± 0.1
WB10	1488 ± 8	360 ± 5	81 ± 0	174 ± 8	61.6 ± 0.3	14.9 ± 0.2	3.3 ± 0.0	7.2 ± 0.3
WB11	1365 ± 6	303 ± 3	26 ± 1	164 ± 6	63.8 ± 0.3	14.1 ± 0.1	1.2 ± 0.0	7.6 ± 0.2
WB12	1271 ± 12	273 ± 4	64 ± 0	162 ± 2	65.1 ± 0.6	14.0 ± 0.2	3.3 ± 0.0	8.3 ± 0.1
WB13	1649 ± 10	392 ± 1	73 ± 1	154 ± 0	63.1 ± 0.4	15.0 ± 0.0	2.8 ± 0.0	5.9 ± 0.0
WB14	1786 ± 8	377 ± 3	46 ± 0	142 ± 1	71.0 ± 0.3	15.0 ± 0.1	1.8 ± 0.0	5.7 ± 0.1
WB15	1951 ± 13	393 ± 4	88 ± 0	182 ± 1	66.7 ± 0.4	13.5 ± 0.1	3.0 ± 0.0	6.2 ± 0.0
Mean (*n* = 15)	1491 ± 184 ^a^	339 ± 36 ^a^	68 ± 21 ^b^	157 ± 15 ^a^	63.3 ± 3.2 ^a^	14.4 ± 0.7 ^a^	2.9 ± 0.9 ^b^	6.7 ± 0.8 ^b^

NSPs, non-starch polysaccharides; AX, arabinoxylan; AG, arabinogalactan; BG, β-glucan; MP, mannose polymers calculated by mannan; BB, barley malt beer; WB, wheat beer. Values are mean ± standard deviation of three replications. Means represent average value ± standard deviation of all beer samples in the same category; different letters indicate significant difference among the average values of barley malt beers and wheat beers under the *p* < 0.05 level.

**Table 4:** The ratio of arabinose to xylose (A/X) and the average degree of polymerization (avDP) of arabinoxylan.

Beer *No.*	Brewers’ Country	Raw Material	A/X	avDP
BB1	Germany	Barley malt	0.71 ± 0.00	46 ± 0
BB2	Germany	Barley malt	0.69 ± 0.01	45 ± 0
BB3	China	Barley malt	0.68 ± 0.00	38 ± 0
BB4	Germany	Barley malt	0.68 ± 0.01	38 ± 0
BB5	Germany	Barley malt	0.71 ± 0.00	48 ± 0
BB6	Germany	Barley malt	0.72 ± 0.00	42 ± 0
Mean (*n* = 6)			0.70 ± 0.02 ^a^	43 ± 4 ^b^
WB1	China	Barley malt, wheat malt	0.61 ± 0.00	40 ± 0
WB2	Germany	Wheat malt, barley malt	0.59 ± 0.00	52 ± 0
WB3	Germany	Wheat malt, barley malt	0.58 ± 0.00	57 ± 0
WB4	Germany	Wheat malt, barley malt	0.58 ± 0.01	51 ± 0
WB5	Germany	Wheat malt, barley malt	0.58 ± 0.00	50 ± 0
WB6	Germany	Wheat malt, barley malt	0.57 ± 0.00	38 ± 1
WB7	Germany	Wheat malt, barley malt	0.58 ± 0.00	57 ± 0
WB8	Germany	Wheat malt, barley malt	0.60 ± 0.00	64 ± 1
WB9	Germany	Wheat malt, barley malt	0.60 ± 0.00	44 ± 0
WB10	Germany	Wheat malt, barley malt	0.57 ± 0.00	55 ± 0
WB11	China	Barley malt, wheat malt	0.66 ± 0.00	61 ± 0
WB12	China	Barley malt, wheat malt	0.60 ± 0.00	50 ± 0
WB13	Germany	Wheat malt, barley malt	0.59 ± 0.00	39 ± 0
WB14	Germany	Wheat malt, barley malt	0.59 ± 0.00	63 ± 0
WB15	Germany	Wheat malt, barley malt	0.57 ± 0.00	83 ± 1
Mean (*n* = 15)			0.59 ± 0.02 ^b^	54 ± 12 ^a^

A/X, the ratio of arabinose to xylose; avDP, the average degree of polymerization; BB, barley malt beers; WB, wheat beers. Values are mean ± standard deviation of three replications. Means represent average value ± standard deviation of all beer samples in the same category; different letters indicate significant difference among the average values of barley malt beers and wheat beers under the *p* < 0.05 level.

**Table 5:** Pearson correlation coefficient matrix of variables for all beer samples.

	AX	AG	BG	MP	NSPs	TC
AX	1					
AG	0.959 **	1				
BG	−0.726 **	−0.695 **	1			
MP	0.646 **	0.683 **	−0.518 *	1		
NSPs	0.979 **	0.966 **	−0.674 **	0.670 **	1	
TC	0.552 **	0.580 **	−0.163	0.276	0.624 **	1
OE	0.392	0.328	−0.184	0.353	0.350	0.221
RE	0.643 **	0.586 **	−0.372	0.318	0.681 **	0.771 **
ABV	0.211	0.158	−0.068	0.290	0.144	−0.056
Viscosity	0.768 **	0.658 **	−0.349	0.568 **	0.765 **	0.510 *
RDF	−0.278	−0.278	0.224	0.023	−0.360	−0.587 **

The parameters are presented as follows: AX, arabinoxylan; AG, arabinogalactan; BG, β-glucan; MP, Mannose polymers; NSPs, non-starch polysaccharides; TC, total carbohydrate; OE, original extract; RE, real extract; ABV, alcohol by volume; RDF, real degree of fermentation. The number of samples *n* = 21, including 15 wheat beers samples and 6 barley malt beers. Data are correlation coefficient *r*; * the correlation coefficient is significant at level *p* < 0.05, and ** the correlation coefficient is significant at the level *p* < 0.01.

**Table 6:** Rotated principal component loadings of variables and evaluating indices.

	PC1	PC2	PC3
Original extract	0.185	0.309	**0.917**
Real extract	0.294	**0.883**	0.199
Alcohol by volume	0.108	0.005	**0.988**
Real degree of fermentation	0.527	0.566	0.399
Viscosity	−0.128	−0.616	**0.747**
Total carbohydrates	0.207	**0.898**	−0.088
Non-starch polysaccharides	**0.835**	0.513	0.056
Arabinoxylan	**0.860**	0.444	0.115
Arabinogalactan	**0.868**	0.406	0.056
β-Glucan	**−0.859**	−0.025	0.064
Mannose polymers	**0.786**	0.069	0.238
Eigenvalue	4.014	3.013	2.662
Explained variance (%)	36.5	27.4	24.2
Cumulative (%)	36.5	63.9	88.1

Note: the first three factors (eigenvalues over 1) are selected for the principal component interpretation; bold numbers are factor loadings higher than 0.60.
